# Supplementary material for: Identification of photoperiod-induced specific miRNAs in the adrenal glands of Sunite sheep (Ovis aries)
Source: Front Vet Sci. 2022 Jul 22;9:888207. doi: 10.3389/fvets.2022.888207 (PMC9354845; doi:10.3389/fvets.2022.888207)
Supplement: Supplementary file 1 [file Data_Sheet_1.ZIP › Supplementary file/miRDeep2 file.zip!/miRDeep2 file/Novel_Structure/SPLP42A2/Novel_549.pdf]

Provisional ID	:	NC_019459.2_4368
Score total	:	2.2
Score for star read(s)	:	-1.3
Score for read counts	:	0
Score for mfe	:	1.9
Score for randfold	:	1.6
Score for cons. seed	:	
Total read count	:	13
Mature read count	:	12
Loop read count	:	0
Star read count	:	1

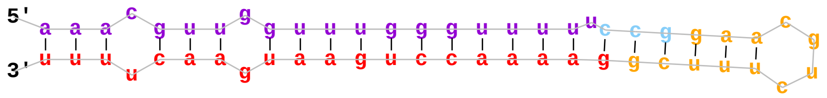

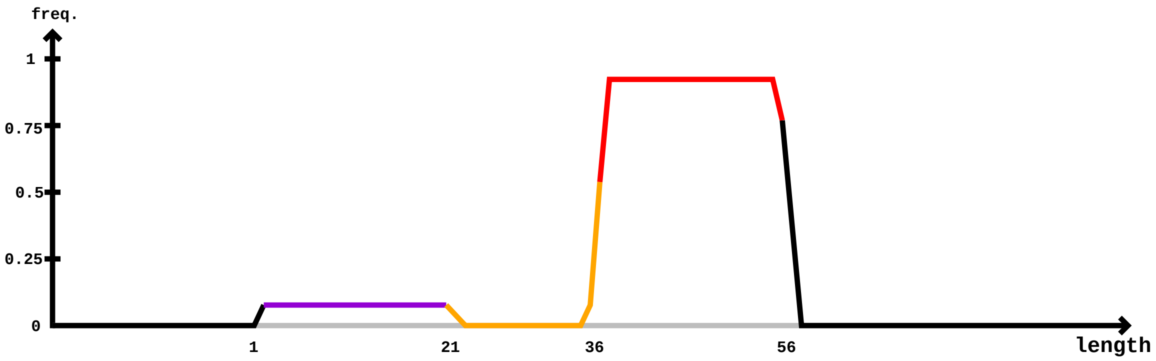

Star **Mature**

```
5'- cacugaguauagguuagcccaaacguugguuuggguuuuuccggaacgucuuucggaaaaccugaaugaacuuuugggcccacccaauacuauggauucccccacugaaa  -3'   obs
    cacugaguauagguuagcccaaacguugguuuggguuuuuccggaacgucuuucggaaaaccugaaugaacuuuugggcccacccaauacuauggauucccccacugaaa         exp
    .....(((((.(((..(((((((.(((.(((((((((((.((((((....)))))))))))))))))).))).)))))))..)))..))))).(((......)))......   reads   mm   sample
    ....................aaacguugguuuggguuuuu......................................................................   1       0    seq
    ......................................................ggaaaaccugaaugaacCuu....................................   1       1    seq
    .......................................................gaaaaccugaaugaacCuuu...................................   6       1    seq
    ........................................................aaaaccAgaaugaacuuu....................................   1       1    seq
    ........................................................aaaaccAgaaugaacuuuu...................................   1       1    seq
    ........................................................aaaaccugaaugaacuuuug..................................   1       0    seq
    ........................................................aaaaccCgaaugaacuuuug..................................   2       1    seq
```
